# Development and Evaluation of Gluten-Free Rice Biscuits: Impact on Glycaemic Index and Bioactive Compounds

**DOI:** 10.3390/foods14132276

**Published:** 2025-06-26

**Authors:** Cristiana L. Pereira, Inês Sousa, Cristina Roseiro, Manuela Lageiro, Vanda M. Lourenço, Carla Brites

**Affiliations:** 1 National Institute for Agricultural and Veterinary Research (INIAV), I.P., Av. da República, 2780-157 Oeiras, Portugal; 2 Department of Earth Sciences, NOVA University of Lisbon, 2829-516 Caparica, Portugal; 3 GREEN-IT Bioresources for Sustainability, ITQB NOVA, Av. da República, 2780-157 Oeiras, Portugal; 4 GeoBioTec Research Center, NOVA FCT, NOVA University of Lisbon, 2829-516 Caparica, Portugal; 5 Department of Mathematics, NOVA Math, NOVA FCT, NOVA University of Lisbon, 2829-516 Caparica, Portugal

**Keywords:** rice bran, γ-aminobutyric acid, γ-oryzanol, phytic acid, estimated glycaemic index

## Abstract

Biscuits are widely consumed snacks traditionally made from wheat flour, which poses challenges for individuals with gluten intolerance and/or diabetes due to their high glycaemic index (GI). This study explored the production of gluten-free biscuits using rice flour from two varieties, Type III (Basmati) and Ariete (Long A), incorporating varying proportions of rice bran as a substitute for milled and brown rice flour. Results show that biscuits made with rice bran had lower starch digestibility and reduced GI (57.06–62.75) compared to control biscuits (66.23–66.95). Rice bran also increased bioactive compounds, such as phytic acid (0.16 to 1.96 g/100 g), γ-oryzanol (0.20 to 86.56 mg/100 g), and γ-aminobutyric acid (6.78 to 16.23 mg/100 g), known for their benefits to diabetes metabolism. Physicochemical analysis further revealed higher protein (6.49%) and lower starch content (30.07%) in rice bran biscuits than in control biscuits (4.20% and 47.38%, respectively). The control biscuits exhibited the highest spread ratio (5.90 and 6.35) and the Ariete variety produced less brittle biscuits (168.30 N), although the addition of bran increased brittleness under cutting force (54.55 N). Sensory evaluation of four rice biscuit formulations showed no significant differences in consumer preferences, regardless of flour type, bran proportion, or rice variety. Among the formulations, the Type III biscuits with an equal blend of milled flour and rice bran stood out, offering improved nutritional quality and a promising option for gluten-free, low-GI diets for consumers seeking healthier alternatives. This formulation also proved a strong balance across key nutritional and bioactive parameters, when compared to a commercial wellness biscuit.

## 1. Introduction

Biscuits are a popular and widely consumed bakery snack, appreciated globally for their convenience and long shelf life [1]. Their enduring popularity aligns with growing consumer demands for ready-to-eat foods that fit into busy lifestyles. In general, biscuits are made from a blend of flour, sugar, and fats [2].

From 2015 to 2020, the prevalence of chronic diseases such as diabetes, cardiovascular disorders, and obesity has raised global health concerns [3,4], which are the leading global cause of death, accounting for just over 70% [4]. This has driven consumers to seek healthier food options that can help prevent or manage these diseases [5,6]. Conventional biscuits, often made from wheat flour, present challenges for individuals with gluten intolerance [7] and are generally unsuitable for people with diabetes due to their high glycaemic index.

Gluten-free flours have emerged as a viable alternative to meet the needs of health-conscious consumers. However, replacing or reducing traditional ingredients in biscuits often alters sensory and physical properties, such as texture and structural integrity, potentially impacting overall sensory quality and consumer acceptance—key factors for market success [8,9]. As a result, alternative cereals have gained importance in reformulating biscuits to optimize their nutritional profile and align with current health trends [10,11,12,13].

Rice is a naturally gluten-free carbohydrate widely consumed worldwide and has increasingly been used in baking experiments as a partial or complete substitute for wheat flour [14,15,16]. Rice varieties vary in starch digestibility [17], with estimated glycaemic index (eGI) values ranging from 65.45 to 77.98. Understanding their physicochemical properties allows for the development of baking formulations with a lower eGI.

In vitro studies of eGI have gained significance as they simulate the digestion process, helping to distinguish between starches that are more or less digestible [18]. According to World Health Organization guidelines [19], foods with a GI below 55 are classified as low-GI, while those above 70 are considered high-GI. The variation in eGI in rice can be attributed to factors such as variety, amylose content, dietary fiber, grain size, form, and cooking properties [17,20].

Most rice varieties have a high GI (>70), but varieties with higher amylose (>25%) and fat content tend to have lower GI scores [21,22]. Additionally, eGI is inversely related to resistant starch (RS) content and rice gelatinization temperature. Basmati varieties, for instance, have higher RS levels and lower eGI compared to other rice types [17].

Since starch is the primary component in biscuits, it significantly influences eGI, along with sugar. As a result, replacing starch with non-starchy or resistant starch ingredients has been explored [7,23,24].

Rice bran and brown rice have been studied for their potential to naturally enhance the nutritional and bioactive content of rice-based foods [25,26,27]. As a by-product of the rice milling industry, rice bran is rich in dietary fiber and protein, while also reducing digestible starch, making it a promising ingredient for healthier biscuit formulations [7]. Furthermore, rice bran contains higher levels of bioactive compounds compared to milled rice [28], including phytic acid, γ-aminobutyric acid, γ-oryzanol, amino acids, and phenolic compounds, which have demonstrated bioactive properties, particularly in relation to metabolic processes associated with diabetes [28,29,30,31].

In vitro and in vivo studies have shown that the bioactive compounds in rice can positively influence the glycaemic index (GI) and glycaemia when consumed through food or supplementation [32,33,34]. These compounds affect starch digestion enzyme activity [35,36,37] and have beneficial effects on metabolic disorders at the cellular level, particularly in managing type II diabetes. These effects include controlling insulin secretion from the pancreas [38,39], by suppressing endoplasmic reticulum stress, and protecting β-cells from apoptosis [40]. Additionally, these bioactive compounds contribute to the reduction of circulating advanced glycation end products (AGEs) by approximately 25% and glycated hemoglobin, addressing dysfunctions related to pancreatic β-cells [36,39,40,41].

This study aims to evaluate the incorporation of brown and milled rice flour, as well as rice bran, in varying proportions, into biscuit formulations using two rice varieties (Ariete and Basmati Type III), with 100% milled rice flour as the control. The primary objectives were to compare the different formulations with the control and identify the optimal rice-based biscuit formulation, considering (i) starch digestibility and the lowest eGI, (ii) nutritional composition, (iii) bioactive compound content: γ-aminobutyric acid (GABA), γ-oryzanol (ORY), and phytic acid (PA), and (iv) the final texture of the biscuit. Additionally, the sensory characteristics of the rice bran-incorporated biscuits were assessed through sensory evaluation by a trained panel. The optimal biscuit formulation must attend to the minor eGI, higher bioactive compounds levels, and a better nutritional composition with higher PRTD levels and lower TS, and with a texture similar to the respective control. Finally, it should be the best positioned in the sensory analysis ranking of the final formulations.

## 2. Materials and Methods

**Figure 1:**
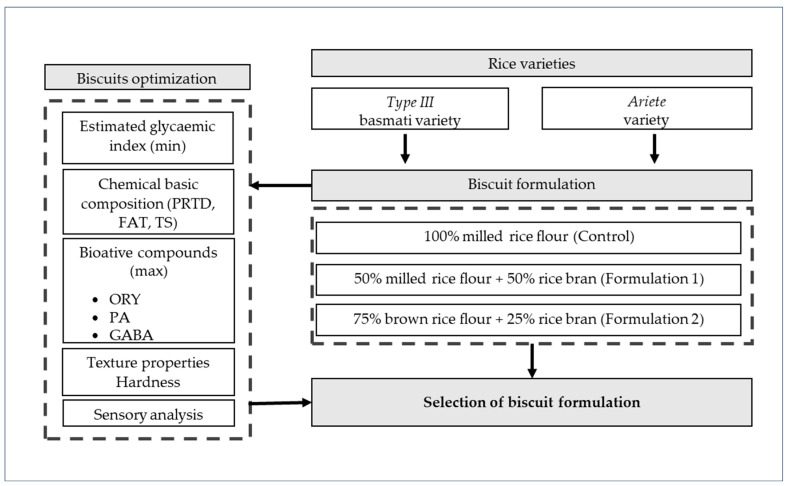
Schematic overview of the biscuit formulations and optimization tests.

Different rice-based biscuits were developed, testing two rice varieties, Ariete (long A grain) and Type III (basmati grain), with different matrices (milled and brown rice flours) and varying rice bran quantities (25% and 50%). The control biscuits were made with 100% rice milled flour. These varieties were selected for their distinct physicochemical properties, and the schematic overview of the biscuit formulations and optimization tests is shown in Figure 1.

Formulations were designed to create relevant variations, with fixed ingredients (butter, water, sugar, and salt). Each formulation was evaluated based on intrinsic parameters, including eGI, starch digestion properties, and bioactive compounds (ORY, GABA, PA). Biscuit quality parameters, such as texture (hardness), were measured, and sensory tests were conducted on the rice bran biscuit formulations. The ideal formulation will be the one with optimized results across all parameters, compared with a commercial wellness biscuit (WB) (Continente, Porto, Portugal). The formulation of this biscuit incorporates wholegrain flours, protein, and fiber: whole wheat flour (41%, oat flakes (14%), stabilized brown rice flour (12%), vegetable protein (5.2%), vegetable fiber (5.2%), puffed millet (3%) and features reduced levels of sugar and fat (sugar cane molasses (12%) and high oleic sunflower oil (12%)).

### 2.1. Rice Biscuit Formulation

The biscuits were prepared following AACC International Method 10-50.05 [42], with modifications to the ingredients. Control biscuits were prepared using 100% rice milled flour from the Ariete (100%AMF) or Type III (100%TMF) varieties. Composite biscuits incorporated either a 50:50 mixture of milled rice flour and rice bran (50%AMF + 50%RB, 50%TMF + 50%RB) or a 75:25 mixture of brown rice flour—Ariete (ABF) or Type III (TBF)—and rice bran (75%ABF + 25%RB, 75%TBF + 25%RB).

The biscuit preparation began by mixing cream shortening, white sugar, and salt. Water, rice flours, and rice bran were then incorporated into the mixture by the food processor (Vorwerk Thermomix TM 31, Vorwerk & Co. Thermomix GmbH, Wuppertal, Germany). The biscuit batter was portioned into 18 g portions and manually shaped into circular forms. The biscuits were baked at 205 °C for 15 min, in an oven (26965, PAULE, Lyon, France). After cooling for 30 min at room temperature, the biscuits were vacuum-packed for further analysis.

### 2.2. γ-Oryzanol Extraction and Quantification

The γ-oryzanol (ORY) extraction was carried out with isopropanol according to the procedures reported by Castanho, et al. (2019) [43], and the dry residue was dissolved with isopropanol. The ORY separation and quantification were performed according to the method reported by Lageiro et al. (2020) [44] at an HPLC system with an Alliance separation module 2690 and a photodiode array detector, PDA 2996 (Waters, Milford, MA, USA) using an ACN:MeOH, (50:50 *v*/*v*) mobile phase at 1.2 mL/min flow rate, 20 μL injection volume and 30 min run time. The separation of ORY compounds was achieved at 35 °C with a reverse phase C18 separation column (Spherisorb ODS2, 4.6 mm × 250 mm, 5 μm, Waters, Milford, MA, USA), and samples were kept at 25 °C till injection. The ORY peaks identification was made at 325 nm by retention times and spectra comparisons with the ones of the ORY standard peaks (TCI Europe, Zwijndrecht, Belgium). The quantification was based on an external calibration curve with ORY standard solutions (10 to 900 mg/L). A linear regression between the total ORY area (peak area sum of the different ORY compounds’ peak areas) and the ORY content in mg/mL was obtained as by [44] with a determination coefficient of 0.9995. The ORY data were expressed in mg/100 g of sample.

### 2.3. Phytic Acid Extraction and Quantification

Phytic acid (PA) content was determined using an enzymatic method. Rice biscuits (~0.4 g) were extracted with a 0.66 M HCl solution, stirring overnight at room temperature. The supernatant (0.5 mL) was then neutralized with 0.75 M NaOH (0.5 mL) and analyzed using the Megazyme K-PHYT kit (Megazyme International, Wicklow, Ireland) according to the manufacturer’s protocol, which measures inorganic phosphorus (Pi) and phytic acid through enzymatic reactions (40 °C) involving phytase and alkaline phosphatase. A subsequent colorimetric reaction between Pi and ammonium molybdate produced a molybdenum blue solution, which was quantified via UV/vis spectroscopy at 655 nm, and the content of free and total inorganic phosphorus was measured. The concentration of PA of each sample was calculated based on the proportional relationship between phosphorus and molybdenum blue using an external calibration curve prepared with dilutions of standard phosphorus (0.5 to 7.5 μg/mL). The content of free and total phosphorus was obtained from each sample, and the difference between these two values was the binding phosphorus. The calculation of PA content assumes that the amount of phosphorus measured is exclusively released from phytic acid and that this comprises 28.2% of PA.

### 2.4. γ-Aminobutyric Acid Extraction and Quantification

The samples (≈1 g) were subjected to acidic hydrolysis in 6 M HCl, at 110 °C for 24 h, before analysis of the amino acid profile [45]. The contents of GABA were determined as derivatives of OPA according to the procedure of Roseiro et al. (2024) [45]. The analysis was performed using an HPLC system equipped with an Alliance Separation Module 2695 with a 2475 fluorescence detector (Waters, Milford, MA, USA). Chromatographic separation was performed using a reverse phase Spherisorb ODS2 column (4.6 mm × 250 mm, 5 μm) (Waters, Milford, MA, USA) at room temperature with a mobile phase consisting of solvent A—0.1 M sodium acetate:methanol: tetrahydrofuran (905:90:5) and solvent B—methanol, employing gradient elution. The gradient changed from 0 to 25% of solvent B in 20 min and from 25 to 100% in 50 min at a flow rate of 1.0 mL/min. GABA was detected by fluorescence with a fluorescence detector at 340 nm (excitation)/425 nm (emission), at the retention time of 15.8 min. Quantification was based on the external standard technique, from a standard curve of peak area vs. concentration of standard GABA (0.02 to 0.1 mg/mL).

### 2.5. Estimated Glycemic Index by In Vitro Enzymatic Method

The biscuits’ digestible starch fractions and the resistant starch fraction (RS) were determined using a digestible starch and resistant starch assay kit K-DSTRS from Megazyme (Wicklow, Ireland), allowing also to quantify the rapidly and slowly digestible starch fractions (RDS and SDS). Then, the eGI was obtained through the kinetic parameters of starch digestion, according to described by Pereira et al. 2023 [17].

### 2.6. Physical Characteristics of Biscuits: Texture and Spread Ratio

Biscuit hardness was assessed by measuring the cutting strength using a sharp-blade probe (HDP/BS blade, 6 cm long and 1 mm thick) on a TA.HD Plus texture analyzer (Stable Micro Systems, Godalming, UK) equipped with a 25 kg load cell in compression mode. The test parameters included a pre-test speed of 1.5 mm/s, test speed of 2 mm/s, post-test speed of 10 mm/s, and a 5 mm cutting distance [46]. The absolute peak force from the force–deformation curve was recorded as the cutting strength [47,48] and analyzed using the BIS2/kB method in Texture Exponent 32 software (version 4.0.11.0, Stable Micro Systems).

The biscuit spread ratio (SR) was determined according to AACC Method 10-50.05 [42] by dividing the biscuit’s diameter by its thickness after baking.

### 2.7. Sensory Analysis of Rice Bran Biscuits

A ranking test (ISO 8587, 1988) [49] was conducted to determine consumer preferences. This forced-choice test evaluates the intensity of sensory attributes across multiple products (Li et al., 2017) [50]. In this study, sensory evaluations were performed on four new biscuit formulations containing 25% and 50% rice bran by a trained panel of 12 panelists (66.7% female and 33.3% male, and the age ranged from 25 to 65 years old). The sensory attributes evaluated included aroma intensity, appearance, texture, flavour, and overall quality. The four biscuit formulations were presented individually in random order. Each panel ranked four biscuits according to their preferences. The scale applied based on intensity for each attribute, with rankings ranging from “1” (lowest intensity) to “4” (highest intensity). The data obtained from the sensory evaluations were analyzed using a non-parametric Friedman test to interpret the organoleptic results.

### 2.8. Statistical Analysis

Each biscuit variant was analyzed using three distinct independent samples, and a two-way ANOVA with mean comparisons assessed the variability of parameters across all biscuit formulations. The Tukey HSD post hoc test was applied to identify significant differences among the formulations. Because validating the assumptions of normality and homogeneity of variances is challenging given such a small sample size, no formal tests for normality or homogeneity of variances were conducted. Instead, a dual approach was implemented to strengthen the reliability of the findings and support the validity of the results. In addition to the parametric ANOVA, the non-parametric Kruskal–Wallis test was performed to compare group differences without relying on assumption-based conditions. Dunn’s post hoc test was then used for pairwise comparisons following Kruskal–Wallis. This combined approach ensures a more robust analysis by considering both parametric and non-parametric perspectives, allowing for comparison of results and increasing the confidence in the conclusions despite the sample size limitations. Tukey’s HSD assumes that the data are normally distributed and that variances are homogeneous, which may not always hold in small samples, potentially making the test more sensitive and prone to detecting significant differences. In contrast, Dunn’s test is a non-parametric method that does not rely on these assumptions, making it more robust in situations where normality or homogeneity of variance may be in question. However, non-parametric tests like Dunn’s are generally less powerful than parametric tests and might detect fewer significant differences, as they tend to be more conservative when dealing with small sample sizes. To further investigate the relationships between variables and samples, a principal component analysis (PCA) was conducted.

For the sensorial analysis, the Friedman test was applied to assess differences in sensory ratings across biscuit variants. The Friedman non-parametric test, used to detect differences in performance across multiple methods, is particularly suited for repeated measures or blocked designs—as in this study, where multiple judges rated each biscuit variant. This test accounts for within-subject variability by considering the differences between the biscuit formulations across all judges. In addition to the Friedman test, the Iman–Davenport test was also used to analyze potential differences between biscuit variants. The Iman–Davenport test is an alternative non-parametric method, specifically designed for situations where assumptions of homogeneity of variance across blocks (judges, in this case) may not hold. It is particularly useful when comparing multiple related groups, especially when the data is subject to block effects that could influence the results. The key difference between the Iman–Davenport test and the Friedman test lies in how they handle the data structure: while both tests are non-parametric, the Iman–Davenport test is more flexible in dealing with heterogeneity in variances, providing a more robust solution in scenarios where variances are unequal across blocks. If either the Friedman or Iman–Davenport test returns a significant result, post hoc pairwise comparisons—typically via the Nemenyi test—are performed.

Statistical analyses were performed at a 5% significance level for the ANOVA, Kruskal–Wallis, Friedman, and post hoc tests. All computations, including modeling first-order kinetic equations for glycaemic index estimation, were carried out using R software (version 4.3.1).

## 3. Results and Discussion

In this study, different biscuit formulations were prepared using combinations of brown and white rice flours with rice bran, to create formulations aimed at reducing the eGI and optimizing the nutritional, bioactive, and sensory value. For the effect, two rice varieties were also selected, Ariete and Type III varieties, with distinct viscosity profiles, starch digestibilities, nutritional properties, and even the aromatic attributes of Basmati rice, which could enhance biscuit sensory attributes.

The results from both the ANOVA and Kruskal–Wallis tests indicated, at the 5% significance level, that there was at least one significant difference in the means across all compounds when comparing the different biscuit formulations. However, when comparing pairwise mean differences between these biscuit groups, some disagreement was observed between the Tukey HSD and Dunn’s tests. Tukey’s test revealed a larger number of significant differences, while Dunn’s test detected fewer significant differences overall, being more conservative. Notably, all the differences detected by Dunn’s test were also identified by Tukey’s test.

The differences between the parametric (Tukey HSD) and non-parametric (Dunn’s) tests can be attributed to the underlying assumptions and sensitivity of these tests, especially given the small sample size of 3 per group.

All tables report pairwise comparisons based on Tukey’s test, with findings also discussed in light of Dunn’s test to provide a more comprehensive understanding of the significant differences across the biscuit formulations. Full results are available in the Supplementary Materials. Throughout the discussion of results and in the Supplementary Materials, the following identifications are used for the biscuits according to the proportion of flour and bran: 100% AMF—rice biscuit control with 100% Ariete milled flour (A0); 50%AMF + 50%RB—50% Ariete milled flour with 50% rice bran (A1); 75%ABF + 25%RB—75% Ariete brown flour with 25% rice bran (A2); 100%TMF—rice biscuit control with 100% Type III milled flour (B0); 50%TMF + 50%RB—50% Type III milled flour with 50% rice bran (B1); and 75%TBF + 25%RB—75% Type III brown flour with 25% rice bran (B2).

**Figure 2:**
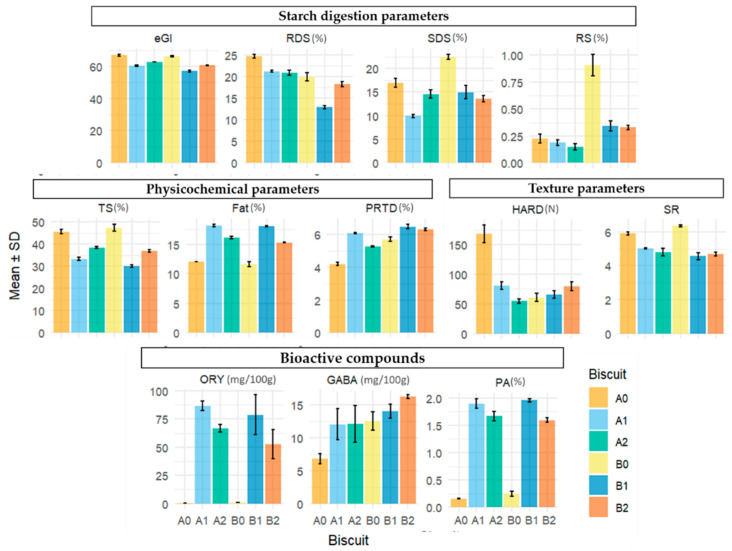
Mean concentration of each compound eGI—estimated glycaemic index; RDS (%)—rapidly digestible starch; SDS (%)—slowly digestible starch; RS (%)—resistant starch; TS (%)—total starch content; FAT (%)—fat content; PRTD (%)—protein content; SR—spread ratio; HARD (N)—hardness; PA (g/100 g)—phytic acid; ORY (mg/100 g)—γ-oryzanol, GABA (mg/100 g)—γ-aminobutyric acid (± SD) by rice biscuit formulations: (A0) control with 100% Ariete milled flour; (A1) 50% Ariete milled flour with 50% rice bran; (A2) 75% Ariete brown flour with 25% rice bran; (B0) control with 100% Type III milled flour; (B1) 50% Type III milled flour with 50% rice bran; (B2) 75% Type III brown flour with 25% rice bran.

The mean values and associated standard deviations of each compound across biscuit formulations are presented graphically in Figure 2, with the corresponding numerical summaries detailed in Table 1, Table 2, Table 3 and Table 4.

### 3.1. Estimated Glycaemic Index and Starch Digestion Fractions of Biscuits

In this study, biscuits were prepared using combinations of brown and white rice flours with rice bran and two different rice varieties, Ariete (Long A grain) and Type III (Basmati grain). Previous study [17] on these varieties revealed differences in their digestibility parameters: Ariete had the highest eGI (74.09), with a rapidly digestible starch (RDS) content of 24.91% and a low resistant starch (RS) content of 0.38%. In contrast, Type III exhibited a lower eGI (67.52), a reduced RDS content of 15.79%, and a higher RS content of 2.53%, making it an interesting candidate for healthier biscuit formulations.

**Figure 3:**
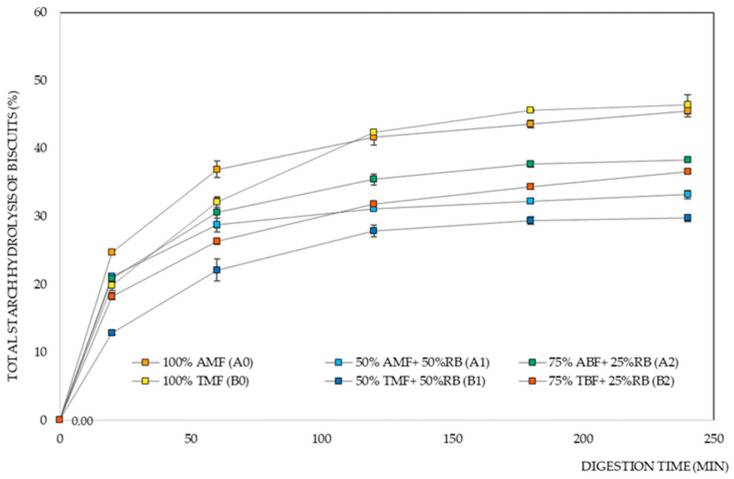
Total starch hydrolysis of rice biscuit formulations. AMF—Ariete milled flour, ABF—Ariete brown flour, TMF—Type III milled flour, TBF—Type III brown flour, RB—rice bran.

Figure 3 shows the starch digestion curves of the different biscuit formulations. We observe that biscuits made with the Ariete variety initially exhibit a faster starch hydrolysis rate when compared to those made with the Type III variety.

Table 1 displays the means and standard deviations for the eGI and starch digestion parameters (RDS, SDS, and RS), along with the results of Tukey’s test for the comparison of mean differences between biscuit formulations for all parameters. The corresponding Dunn’s test results, which further evaluate the significance of these differences, are presented in Table S1 of the Supplementary Materials.

For eGI, Tukey’s test indicated significant differences among all biscuit formulations except between the controls (A0, B0) and between A1 and B2 (Table 1). The eGI and starch digestion fractions indicate that the lowest eGI (57.06) was observed in the formulation containing 50%TMF + 50%RB (B1). In contrast, the control formulations without added bran (A0, B0) had the highest eGI values (66.95 and 66.23, respectively), reinforcing the beneficial role of rice bran in lowering eGI when replacing part of the rice flour. According to Dunn’s test, the only significant differences observed were between the control biscuits (A0, B0) and the B1 formulation, further strengthening the impact of the 50%TMF + 50%RB blend in reducing eGI.

**Table 1:** Means of eGI and starch digestion parameters for each rice biscuit formulation.

Rice Variety	Biscuit Formulation		eGI	Starch Digestion Properties		
				RDS (%)	SDS (%)	RS (%)
	**100% AMF (control)**	A0	66.95 ± 0.70 a	24.69 ± 0.47 a	16.94 ± 0.98 b	0.22 ± 0.04 bc
**Ariete**	**50% AMF + 50% RB**	A1	60.31 ± 0.32 c	21.18 ± 0.19 b	9.89 ± 0.33 d	0.19 ± 0.03 c
	**75% ABF + 25% RB**	A2	62.75 ± 0.14 b	20.85 ± 0.60 b	14.59 ± 0.86 bc	0.15 ± 0.03 c
	**100% TMF (control)**	B0	66.23 ± 0.47 a	19.87 ± 1.00 bc	22.51 ± 0.54 a	0.91 ± 0.10 a
**Type III**	**50% TMF + 50% RB**	B1	57.06 ± 0.59 d	12.87 ± 0.41 d	14.97 ± 1.41 bc	0.34 ± 0.04 b
	**75% TBF + 25% RB**	B2	60.59 ± 0.10 c	18.24 ± 0.59 c	13.61 ± 0.68 c	0.33 ± 0.02 b

eGI—estimated glycaemic index; RDS—rapidly digestible starch; SDS—slowly digestible starch; RS—resistant starch. AMF—Ariete milled flour, ABF—Ariete brown flour, TMF—Type III milled flour, TBF—Type III brown flour, RB—rice bran. Values in the same column with different letters are significantly different at *p* ≤ 0.05 by HSD Tukey test.

In the case of RDS, the control formulation A0 was declared by Tukey’s test to be significantly different from all other formulations, with the highest value recorded at 24.69%. The 50%TMF + 50%RB formulation (B1) also showed significant differences from all other groups, with the lowest value recorded at 12.87%, highlighting its role in significantly reducing the RDS percentage. Additionally, the results indicate that there are significant differences in RDS content between the Ariete rice bran (A1, A2; higher RS values) and Type III rice bran (B1, B2; lower RS values) formulations. Dunn’s test identified only the difference between A0 and B1 as significant, further emphasizing the distinct impact of B1 in reducing RDS compared to the other formulations.

Tukey’s test for SDS indicates that the 100%TMF control formulation (B0) and the 50% AMF + 50%RB formulation (A1) stand out, respectively, with the highest and lowest SDS content (22.51% and 9.89%) compared to all other groups. In addition, results show that introducing RB into the Type III formulations leads to a reduction in SDS percentage, with B1 and B2 exhibiting lower SDS content compared to B0. Dunn’s test reinforces the significant difference between the lowest (B0) and highest (A1) SDS values and corroborates the significant differences between B0 and B2, further emphasizing the distinction between these two formulations.

Regarding RS content, Tukey’s test reveals that the 100%TMF control formulation (B0) exhibits the highest RS content (0.91%), which is significantly different from all other formulations. A0, A1, and A2 all fall into the same group, indicating no significant differences between these formulations in terms of RS content. B1 and B2 have similar RS content and are significantly different from B0. Additionally, the results indicate that there are significant differences in RS content between the Ariete (A0, A1, A2; lower RS values) and Type III (B0, B1, B2; higher RS values) formulations. Dunn’s test only corroborates the significant difference between A2 and B0, and does not support the other differences observed by Tukey’s test.

These results indicate that some key factors, such as the introduction of non-wheat flours from pseudocereals and no-cereal products, are potential ingredient candidates for low eGI formulations [7,51,52,53], also reducing the starch content available for digestion [7,16,51,53,54].

Figure 3 and Table 1 show the significant influence of the rice variety factor on the eGI of the biscuits, especially in the 50% milled flour and 50% rice bran formulations, where there was a lower digestibility rate for the biscuits with Basmati Type III flour. The type of starch and its different properties also contribute to the differences obtained for RDS and SDS [55], where the results show that the Type III variety stands out as a more slowly digested variety when compared to Ariete. The high eGI is strongly correlated with the rapid gelatinization of a starch, and the lower content of indigestible starch, which is in concordance with the properties obtained in a previous study by Pereira et al. 2023 [17], where Basmati varieties showed higher RS values, and even higher gelatinization temperatures compared to the Ariete variety. The results obtained for the starch digestion parameters of the biscuit formulations suggest that the Basmati rice variety would be the best choice.

### 3.2. Basic Chemical Composition of Biscuits

The basic chemical composition of control and rice bran biscuits is summarized in Table 2, along with the results of Tukey’s test for the comparison of mean differences between biscuit formulations for all chemical parameters. The corresponding Dunn’s test results, which further evaluate the significance of these differences, are presented in Table S1 of the Supplementary Materials.

Results in Table 2 show that the inclusion of rice bran in the Ariete and Type III biscuit formulations reduced the total starch content (TS), thereby lowering the starch available for digestion [54] compared to the control biscuits. This aligns with prior studies highlighting the benefits of incorporating non-starchy ingredients as alternatives [7] (Goubgou et al., 2021).

**Table 2:** Means of basic chemical composition for each rice biscuit formulation.

Rice Variety	Biscuit Formulation		Physicochemical Properties		
			TS (%)	FAT (%)	PRTD (%)
**Ariete**	**100% AMF (control)**	A0	45.68 ± 1.02 a	12.04 ± 0.02 d	4.20 ± 0.11 e
	**50% AMF + 50% RB**	A1	33.38 ± 0.79 c	18.22 ± 0.22 a	6.09 ± 0.04 b
	**75% ABF + 25% RB**	A2	38.42 ± 0.42 b	16.18 ± 0.20 b	5.27 ± 0.05 c
**Type III**	**100% TMF (control)**	B0	47.38 ± 1.66 a	11.63 ± 0.40 d	5.70 ± 0.14 d
	**50% TMF + 50% RB**	B1	30.07 ± 0.63 d	18.10 ± 0.11 a	6.49 ± 0.14 a
	**75% TBF + 25% RB**	B2	36.91 ± 0.50 b	15.34 ± 0.05 c	6.31 ± 0.08 ab

TS—total starch content; FAT—fat content; PRTD-protein content; AMF—Ariete milled flour, ABF—Ariete brown flour, TMF—Type III milled flour, TBF—Type III brown flour, RB—rice bran. Values in the same column with different letters are significantly different at *p* ≤ 0.05 by HSD Tukey test.

Additionally, biscuit formulation B1, with the lowest value at 30.07%, is significantly different from all other groups, while A0 and B0 show the highest TS contents (45.68% and 47.38%, respectively). Dunn’s test results only support the significant differences between B1 and the two control groups (A0 and B0).

For FAT content, Tukey’s test reveals that there are no significant differences between the control formulations, A0 and B0. However, both A0 and B0 exhibit the smallest fat percentages, significantly different from all other formulations, which show higher fat contents. Additionally, the inclusion of rice bran in each rice type (Ariete or Type III) led, as expected, to an increase in fat content, with significant differences observed between A0 and both A1 and A2, as well as between B0 and both B1 and B2. Dunn’s test results corroborate the significant difference between B0 and B1.

Regarding PRTD, a distinction emerged between the rice varieties. Biscuits made with the Type III variety generally exhibited higher PRTD content compared to those made with the Ariete variety. However, no significant differences were observed between A1 (Ariete) and B2 (Type III), indicating that the impact of rice variety on PRTD content was not uniform across all formulations. Dunn’s test revealed significant differences between B1 and B2 (higher PRTD values) and A0 and A2 (lower PRTD values), further emphasizing the contrasting PRTD levels between formulations containing Type III rice and those with Ariete rice. This difference can be attributed to the intrinsic PRTD of the flours used: Type III flours exhibited higher PRTD levels (8.38% in milled flour and 8.96% in brown flour) compared to Ariete flours (6.88% in milled flour and 7.67% in brown flour). Rice bran is characterized by its high lipid (16.9%) and protein content (12.8%)—approximately six and two times higher than the lipid and protein content of milled and brown rice flour, respectively. This increase in FAT and PRTD content positively influenced the eGI by reducing starch digestibility [55,56,57]. A similar trend was observed when comparing the results in Table 1 and Table 2. This can be explained by the interactions and formation of starch–protein and starch–lipid complexes, which can cause restriction of the swelling of starch granules, preventing amylolytic enzymes from accessing glycosidic bonds, and therefore limiting the process of gelatinization and retrogradation of starch, influencing the reduction of eGI [55,58]. The introduction of brown flour and rice bran improved the nutritional value of the new rice biscuit formulations and reduced total sugars (TS). This finding aligns with Bultum et al. (2020) [54], who reported that replacing wheat flour with rice bran in biscuits increased protein and lipid content while reducing carbohydrates.

### 3.3. Texture of Biscuits

Table 3 displays the means and standard deviations for texture parameters (SR and HARD), along with the results of Tukey’s test for the comparison of mean differences between biscuit formulations for all parameters. The corresponding Dunn’s test results, which further evaluate the significance of these differences, are presented in Table S1 of the Supplementary Materials.

The analysis of biscuit hardness values, presented in Table 3, demonstrates how the inclusion of rice bran and the choice of rice variety influence the textural properties of biscuits. Hardness (HARD), defined as the force required to compress a substance between molar teeth, is closely tied to the primary ingredients—rice flour and rice bran.

**Table 3:** Texture properties for each rice biscuit formulation.

Rice Variety	Biscuit Formulation		SR	HARD (N)
	**100% AMF (control)**	A0	5.90 ± 0.08 b	168.30 ± 14.68 a
**Ariete**	**50% AMF + 50% RB**	A1	5.03 ± 0.04 c	80.33 ± 6.24 b
	**75% ABF + 25%RB**	A2	4.81 ± 0.23 cd	54.55 ± 3.28 c
	**100% TMF (control)**	B0	6.35 ± 0.05 a	60.82 ± 6.50 bc
**TypeIII**	**50% TMF + 50% RB**	B1	4.57 ± 0.21 d	65.76 ± 5.94 bc
	**75% TBF + 25% RB**	B2	4.68 ± 0.11 cd	79.71 ± 7.19 b

SR—spread ratio; HARD—hardness, AMF—Ariete milled flour, ABF—Ariete brown flour, TMF—Type III milled flour, TBF—Type III brown flour, RB—rice bran. Values in the same column with different letters are significantly different at *p* ≤ 0.05 by HSD Tukey test.

Table 3 reveals a decreasing trend in hardness means for Ariete-based biscuits, contrasting with an increasing trend in means for Type III-based biscuits. Tukey’s test confirms strong differences between the Ariete levels, indicating that each increment of rice bran significantly reduces hardness. However, for Type III biscuits, no significant differences are found between levels, suggesting a more stable structural profile despite the increasing trend in means. Tukey’s test results also reveal that the mean hardness of the control A0 formulation is statistically different from those of the Type III formulations (B0, B1, and B2), although this difference is not corroborated by Dunn’s test. Dunn’s test, being more conservative, only detects a significant difference between A0 and A2. Interestingly, the difference in means between A0 and A1 is nearly twofold, yet it is not flagged as significant, likely due to the limited number of observations affecting statistical power.

From these results, we can conclude that the viscoelastic/pasting properties of rice variety and rice flour type had a higher influence on the HARD parameter. Kang et al. 2016 [59] found that different rice varieties produced biscuits with different properties. The same happened in the study by Rai et al. (2019) [60], but with wheat varieties.

The rheological characteristics of flours, such as pasting properties and starch gelatinization, critically affect the stiffness and texture of baked goods [50,51]. The results also highlight the significant role of rice variety in determining biscuit hardness and final texture, which may be explained by differences in flour viscosity properties. Previous research indicates that biscuit quality is closely linked to starch characteristics [61,62]. The texture of biscuits does not depend on the protein/starch structure, but mainly on the gelatinization process of starch and supercooled sugars [61,63]. A rapid viscosity analyzer (RVA) is a commonly used tool to evaluate the rheological characteristics of starch and starchy ingredients under controlled temperature conditions [64]. Peak viscosity, for example, reflects the extent of starch granule swelling. Ariete flour displayed higher peak viscosity and a lower gelatinization temperature (3352 cP, 65.4 °C) compared to Basmati Type III flour (1776 cP, 72.7 °C). These parameters differ for the brown flours, with Ariete having a higher viscosity peak and lower gelatinization temperature (2200 cP, 66.9 °C) than the Type III variety (1310 cP, 72.6 °C). The rapid gelatinization of the Ariete variety’s starch and its high peak viscosity may explain the high hardness of the final biscuits compared to the Basmati control. This effect is likely most critical during the first few minutes of baking, as temperatures rise from 25 to 50 °C, causing sugar to dissolve and fat to melt [65]. During this stage, starch granules compete for available water, forming a strong structural network. Since starch granules are insoluble at room temperature and only begin to swell and dissolve as the temperature rises [66], the competition for water during the initial baking phase can be quite challenging. This is then followed by the gelatinization process, where starch granules irreversibly swell upon exposure to water at temperatures above 60–70 °C.

Other intrinsic factors of flours, such as endogenous α-amylase activity, the amylose-to-amylopectin ratio, and structural attributes, play key roles [64,67,68,69] in the different pasting properties of flours. Preliminary results on the intrinsic activity of α-amylase, measured using the stirring number (SN) method for Ariete and Basmati rice varieties, indicate higher α-amylase activity in brown flours (SN Ariete = 234.5; SN Basmati = 184.5) compared to milled white flours (SN Ariete = 145.6; SN Basmati = 149.5). Wang et al. (2021) [67] demonstrated that inactivating α-amylase enhances flour viscosity, while amylopectin chain-length distribution (comprising 70–80% of starch) determines pasting behavior. In our study, adding rice bran had a notable effect on the Ariete variety: replacing rice flour with bran significantly reduced the final hardness of the biscuits. Jia et al. (2020) [51] found that during the baking process, the presence of rice flour delayed the gelatinization of starch, reducing the hardness of the biscuits. Canalis et al. (2019) [70] confirmed a reduction in pasting properties when wheat flour is replaced by different fibers. On the one hand, this change could be due to the fact that there are fewer starch granules available to gelatinize [70], increasing the gelatinization temperature [66]. At the same time, the fibers retain more water than wheat flour, so there is less water available, which would make it difficult for the starch granules to swell [65], and more moisture was retained in the biscuits, resulting in a decrease in the hardness of the biscuits [51]. When the pasting properties of the fiber/wheat flour mixtures were measured, they showed higher viscosity peaks, and reduced breakdown and setback [65]. This means that they had a lower capacity for starch to bind to water, greater dough strength, and lower gelling capacities when compared to the control. In our study, given that the rice variety factor varied, these parameters were evaluated individually for each flour, revealing that although the Ariete variety has a higher peak viscosity than the Type III flour, it has a lower resistance and gelling capacity than the Type III variety. These parameters can help predict different flour behaviors when the bran is added and the baking process occurs, resulting in more or less brittle final biscuits.

Intrinsic properties of the rice bran, namely its nutritional properties, can be determinants for the final hardness of the biscuits. A biscuit is made from a low amount of water, and when new ingredients with high water retention capacities are mixed in, they compete with the constituents of the flour for the available water [65]. The bran introduced has a fiber content of 8.88%, a protein content of 12.80%, and a fat content of 16.86%, higher than the values of the flours used. The study by Chauchan et al. (2016) [71] noted that substituting rice flour with brown rice flour or rice bran decreased biscuit hardness. The formation of amylose–lipid complexes, which hinder starch crystallization and amylose retrogradation, further disrupts gelatinization [54,72,73]. These combined effects highlight the balance between ingredient composition and biscuit textural outcomes.

Regarding physical properties, the addition of 25% and 50% rice bran reduced the extensibility of the final biscuits and consequently spread ratio (SR), consistent with the findings of Sudha et al. (2007) [74]. Tukey’s test results for the spread ratio (SR) show that the control formulations A0 and B0 differ significantly from each other and from all other biscuit formulations, with B0 exhibiting the highest SR. Mixing rice bran with both types of flour contributes to a decrease in SR, which is influenced by the interaction between protein and starch in competing for available water during dough preparation, although there is no significant difference between the mix percentages within each type of flour. Dunn’s test results further confirm the decrease in SR in the Type III formulations from B0 to both B1 and B2, with no significant difference observed between B1 and B2.

### 3.4. Bioactive Compounds

All the bioactive compounds analyzed in this study—PA, ORY, and GABA—offer health benefits and have shown no adverse effects when consumed in market-available supplement doses: 100–380 mg of PA, 20–200 mg of ORY, and 200–600 mg of GABA [75,76,77,78,79,80,81,82]. Their concentrations in rice are influenced by multiple factors, including grain growth and maturation, genetic background, and environmental conditions [83,84]. Additionally, processing variables such as milling degree and pericarp color can contribute to variations in these compounds [85].

Table 4 presents the means and standard deviations for the bioactive compounds, along with Tukey’s test results for comparing the mean differences across the various biscuit formulations. In addition, Dunn’s test results, which offer further insight into the significance of these differences, can be found in Table S1 in the Supplementary Materials. 

**Table 4:** Bioactive compounds analyzed in rice-based biscuit formulations.

Rice Variety	Biscuit Formulation		PA (g/100 g)	ORY (mg/100 g)	GABA (mg/100 g)
	**100% AMF (control)**	A0	0.16 ± 0.01 c	0.20 ± 0.02 c	6.78 ± 0.79 b
**Ariete**	**50% AMF+ 50%RB**	A1	1.90 ± 0.09 a	86.56 ± 4.32 a	12.03 ± 2.35 a
	**75% ABF+ 25%RB**	A2	1.67 ± 0.09 b	66.81 ± 3.52 ab	12.08 ± 2.80 a
	**100% TMF (control)**	B0	0.25 ± 0.05 c	0.92 ± 0.04 c	12.50 ± 1.40 a
**Type III**	**50% TMF+ 50%RB**	B1	1.96 ± 0.03 a	78.78 ± 17.74 a	14.05 ± 1.06 a
	**75% TBF+ 25%RB**	B2	1.59 ± 0.04 b	52.71 ± 12.84 b	16.23 ± 0.30 a

PA—phytic acid; ORY—γ-oryzanol, GABA—γ-aminobutyric acid, AMF—Ariete milled flour, ABF—Ariete brown flour, TMF—Type III milled flour, TBF—Type III brown flour, RB—rice bran. Values in the same column with different letters are significantly different at *p* ≤ 0.05 by HSD Tukey test.

Table 4 shows that PA was the most abundant bioactive compound, with concentrations ranging from 0.16 to 1.96 g/100 g, while ORY levels ranged from 0.20 to 86.56 mg/100 g. The positive impact of rice bran and brown rice flour on bioactive compound concentrations is well-documented [86,87]. Our results align with these findings, confirming that rice bran serves as a valuable natural source of bioactive compounds that enhance the nutritional profile of these biscuits. Similarly, Espinales et al. (2022) [88] reported that replacing wheat flour with rice bran in bread improved the content of these bioactive compounds.

Tukey’s test results for PA show significant differences between biscuit formulations.

Within the Ariete-type biscuit formulations (A0, A1, A2), significant differences were observed in PA content, with formulation A0 showing the lowest level, followed by A2, and A1 exhibiting the highest levels. A similar trend was observed within the Type III formulations (B0, B1, B2), where B0 exhibited the lowest PA levels, B2 showed intermediate values, and B1 demonstrated the highest PA content. The control formulations, A0 and B0, exhibited the overall lowest PA levels, while formulations containing rice bran and brown flour, such as A1 and B1, demonstrated the overall highest levels. Formulations A2 and B2 displayed intermediate values. These results suggest that the inclusion of rice bran and brown flour substantially increased the PA content in both the Ariete-type and Type III biscuit formulations, with varying effects depending on the formulation. However, Dunn’s test results support only that A0 significantly differs from A1 and B1. Interestingly, although there is a substantial increase in PA from B0 to B1, this difference is not declared significant by Dunn’s test. This again highlights a potential issue of statistical power, which may have limited the ability to detect this difference despite its apparent size. These findings confirm that while rice bran and brown flour contribute to increased PA, the magnitude of this effect varies across formulations and may be influenced by statistical sensitivity.

Tukey’s test results for ORY closely follow those observed for PA, with one key exception: the difference between A1 and A2 is not significant. Within the Ariete-type formulations, A0 exhibited the lowest ORY content, while A1 and A2 had higher values, but without a significant difference between them. In the Type III formulations, the same trend observed for PA holds, with B0 showing the lowest ORY levels, B1 the highest, and B2 falling in between. Likewise, Dunn’s test results for ORY are in line with those of PA, with the only significant difference observed between A0 and A1. The significant increase in ORY, as well as phytic acid, compared to the controls, is attributed to the presence of bran in these formulations, where these compounds naturally occur in higher concentrations [28]. These findings are consistent with those reported by Itagi et al. (2023) [89], who observed elevated ORY levels in biscuits containing brown rice flour. Similarly, Souza et al. (2018) [90] confirmed increased ORY content in rice biscuits with the addition of rice bran.

Tukey’s test results for GABA indicate that A0 had significantly lower levels compared to all other formulations (A1, A2, B0, B1, B2), which did not show significant differences among themselves. This suggests that the inclusion of rice bran and brown flour did not lead to significant variations in GABA content across formulations within both the Ariete and Type III groups. Likewise, Dunn’s test results for GABA differ slightly from Tukey’s findings, with the key difference being that A0 only exhibited a significant difference with B2. This suggests that while Tukey’s test identified differences between A0 and all other formulations, Dunn’s test highlights a more limited distinction, specifically between A0 and B2.

GABA, a key inhibitory neurotransmitter in the central nervous system, is also naturally present in many foods, including rice [91]. Although the natural addition of rice bran and brown flour did not substantially increase GABA content in the biscuits, previous studies have demonstrated effective strategies for enhancing GABA levels in foods. For instance, Kim et al. (2015) [92] reported that adjusting moisture content and adding glutamic acid increased GABA concentrations in rice bran from 30 mg to 523 mg/100 g. Other methods, such as germination [93,94] and fermentation with microorganisms [95,96], have also been shown to significantly boost GABA levels. These findings suggest that while our current formulations did not achieve notable increases in GABA, targeted processing techniques could be explored in future studies to enhance its presence in rice bran-enriched biscuits.

### 3.5. Principal Component Analysis

**Figure 4:**
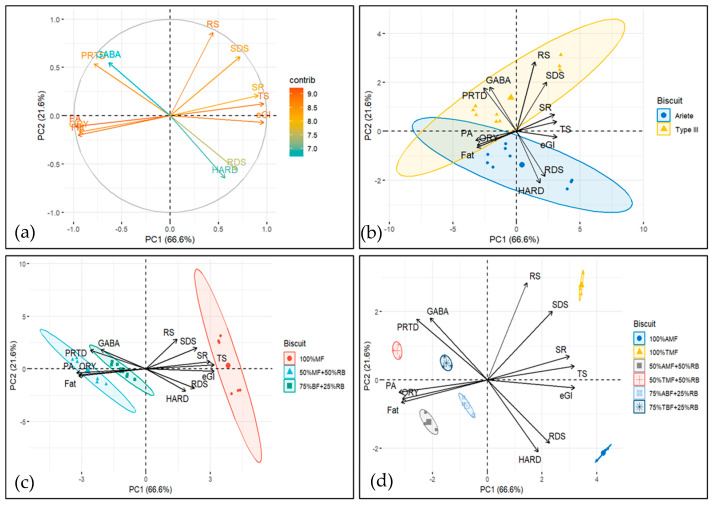
Principal component analysis of data from starch digestion, nutritional, physical, and bioactive compound parameters of rice-based biscuit formulations: (**a**) contributions of variables in accounting for the variability; (**b**) biplot of biscuit rice varieties and variables analyzed; (**c**) biplot of biscuit rice formulations with and without mixing rice bran, and (**d**) biplot of all the formulations and variables analyzed;.

Principal component analysis (PCA) was performed (Figure 4) to integrate the results from various parameters, including starch digestion characteristics (RDS, SDS, RS, eGI), nutritional attributes (PRTD, FAT, and TS contents), physical properties (HARD and SR), and bioactive compounds (ORY, PA, and GABA).

The variables onto the PC1/PC2 plane, the first two principal components explained 88.2% of the total variance, with the first component (PC1) accounting for 66.6% and the second component (PC2) explaining 21.6%. The biplots in Figure 4 suggest that PC1 captures a contrast between bioactive/chemical compounds (GABA, ORY, PA and FAT, PRTD) and starch digestion and texture-related characteristics (TS and RS, RDS, SDS, eGI and SR, HARD). Higher values of PC1 are associated with higher values of starch digestibility and texture properties, and with lower values of bioactive compounds and certain chemical properties (FAT, PRTD). 

PC2 captures a more complex relationship, where bioactive compounds (PA, ORY), chemical compounds (FAT), glycaemic index (eGI), rapid digestible starch (RDS), and texture (HARD) are all positioned on the negative side. This indicates that higher PC2 values correspond to lower levels of these parameters.

Conversely, on the positive side of PC2, we observe starch digestion (RS, SDS), chemical compounds (PTRD, TS), bioactive compounds (GABA), and texture properties (SR), suggesting that higher PC2 values are associated with increased levels of these components.

The biplots in Figure 4b–d display the ellipses for different factors. Figure 4b shows the ellipses for rice type (Ariete vs. Type III; two ellipses). Figure 4c presents the ellipses for each formulation (100% milled flour, 50% milled flour—50% rice bran, and 75% brown flour—25% rice bran; three ellipses). Figure 4d displays the ellipses for each rice type and formulation combination (six ellipses). When compounds and formulation groups, as represented by the ellipses, share the same quadrant, it suggests a positive relationship, meaning the compound is likely to be more abundant or influential in that formulation. The closer the compound’s vector is to the ellipse, the stronger the positive association with the formulation.

The biplot in Figure 4b shows that while Ariete and Type III rice varieties share some common characteristics (as indicated by the overlap in the 2nd and 3rd quadrants), they also exhibit distinct differences in their profiles, which are represented by the unique quadrant crossings. This indicates that each rice type has its own set of influential compounds, which may be relevant when selecting rice types for specific formulations or products.

The biplot in Figure 4c reveals a clear separation among the biscuit formulations within the control, 50%-50% and 75%-25% mix groups, with no overlap between the ellipses. This indicates distinct compositional or functional differences between the formulations, highlighting the unique contributions of each formulation to the overall profile of the biscuits. Notably, the ellipse for the control formulation is positioned on the right (positive side of PC1), while the ellipses for the 50%-50% and 75%-25% mixes are positioned on the left (negative side of PC1). This suggests that the control formulation has distinct characteristics compared to the mixed formulations, which share a more similar profile in terms of the variables captured by PC1. In addition, (i) starch digestion compounds, texture parameter SR and chemical compound TS are higher in the control formulations; (ii) bioactive compounds PA and ORY as well as chemical compound FAT are higher in the 50%-50% mix formulations; and (iii) bioactive compound GABA, chemical compound PRTD, and texture parameter HARD are higher in the 75%-25% formulations. Although HARD is not in the same quadrant as the 75%-25% formulations, its vector is closer to the 75%-25% formulations’ ellipse (which crosses the 2nd, 3rd, and 4th quadrants) than to the control formulations’ ellipse, indicating that HARD is more strongly associated with the 75%-25% formulations.

Figure 4d further illustrates the distinct profiles of the six biscuit formulations, represented by ellipses for the two control formulations, the two 50%-50% mix formulations, and the two 75%-25% mix formulations. The lack of overlap between these ellipses emphasizes the ability of the principal components (PC1 and PC2) to capture the differences between formulations. The positioning along the principal components reveals specific trends: the Ariete control is located in the positive PC1 and negative PC2 quadrant, while the Type III control is in the positive PC1 and positive PC2 quadrant. The 50%-50% formulations are situated in the negative PC1 and negative PC2 quadrant, while the 75%-25% formulations are positioned in the negative PC1 and positive PC2 quadrant. This distribution underscores the influence of formulation type on the measured characteristics.

### 3.6. Comparison with Commercial ”Wellness Biscuit”

**Figure 5:**
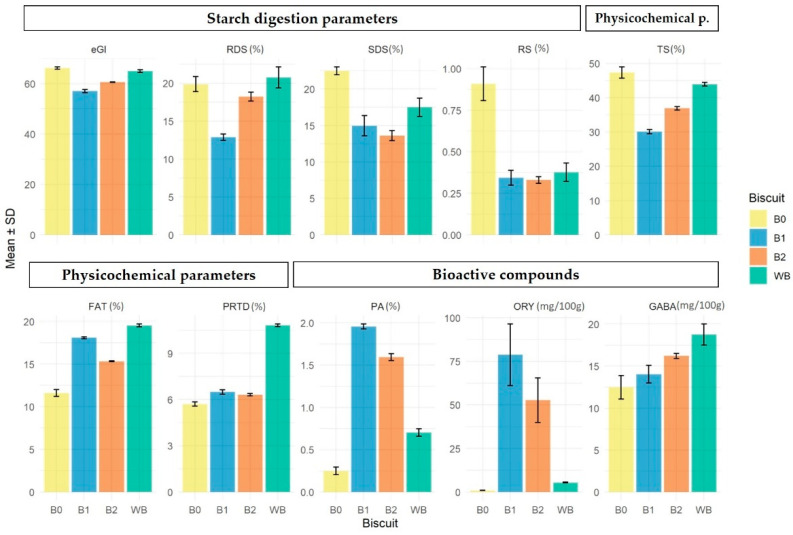
Intrinsic composition (physicochemical parameters, starch digestion parameters, and bioactive compounds of a wellness biscuit (WB), 100%TMF (B0), 50%TMF + 50%RB (B1), 75%TBF + 25%RB (B2) formulations (%—RDS, SDS, RS, TS, FAT, PRTD and PA; mg/100 g—ORY and GABA).

Analysis of the results and PCA indicated that formulations with added rice bran tended to increase bioactive compounds, PRTD, and FAT, while reducing eGI and starch digestibility parameters. Specifically, statistical analysis revealed significant differences between the Type III formulations and the controls. Therefore, starch digestibility, basic nutrient content, and bioactive compound levels for the Type III biscuit formulations were compared with those from a commercially available “Wellness Biscuit” (WB) (1100000094, Continente) (Figure 5).

This commercially available biscuit was developed using a co-creation methodology, incorporating consumer input based on their preferences. The formulation is aligned with contemporary health-conscious eating trends, particularly in terms of ingredients, as it incorporates wholegrain flours, protein and fiber (whole wheat flour (41%), oat flakes (14%), brown rice flour (12%), vegetable protein (5.2%), vegetable fiber (5.2%), puffed millet (3%)) and features reduced levels of sugar and fat. Similar to the aim of this study, the WB is intended to be a nutritionally balanced biscuit that helps moderate the rapid absorption of glucose into the bloodstream (glycaemia).

For the comparison, only intrinsic composition parameters—such as starch digestibility, chemical, and bioactive content—were considered as shown in Figure 5.

The comparison between the WB and the three Type III rice formulations (B0—100% milled flour, B1—50% milled flour and 50% rice bran, B2—75% milled flour and 25% rice bran) across various digestion, bioactive, and chemical parameters revealed several significant differences based on Tukey’s post hoc test (Table 5; Table S2 from the Supplementary Materials).

Formulation B1 exhibited significantly lower values for estimated glycemic index (eGI), rapidly digestible starch (RDS), and total starch (TS), indicating potential benefits in glycemic response and starch content. In contrast, B1 had significantly higher values for phytic acid (PA), and both B1 and B2 showed elevated levels of γ-oryzanol (ORY), with no significant difference between them. The control formulation B0 was associated with significantly higher values of slowly digestible starch (SDS) and resistant starch (RS). The WB had the highest fat (FAT) and protein contents (PRTD), followed by B1, which also showed significantly higher values than the other formulations. Significant differences were observed among the WB biscuit and both B0 and B1 formulations in terms of γ-aminobutyric acid (GABA) content.

Since the goal of this study is to enhance nutritional quality while improving or maintaining functional digestion properties, Formulation B1 appears to be the most promising overall. It demonstrated a strong balance across key nutritional and functional parameters: (i) B1 had significantly lower eGI, RDS, and TS—favorable for glycemic control and reduced starch content; (ii) it showed significantly higher PA and shared the highest ORY values with B1—indicative of improved antioxidant potential; (iii) although WB had the highest FAT and PRTD, B1 was second highest, preserving a good nutritional profile.

By incorporating 50% rice bran—a by-product valued for its health and sustainability benefits—B1 enhances bioactivity, improves glycemic response, and maintains acceptable fat and protein contents. Unless maximizing SDS and RS is a specific target (in which case, B0 might be preferred), B1 emerges as the most balanced and nutritionally enhanced formulation among those tested.

However, it is important to note that these results were primarily supported by Tukey’s test. When using Dunn’s non-parametric test (Table S3 in Supplementary Material), which may have suffered from limited statistical power due to the small sample size, only a few comparisons remained significant—namely, the lower RDS in B1 and the higher FAT and PRTD values in WB compared to B0. This inconsistency highlights the need for cautious interpretation and further validation with larger samples. Nonetheless, the overall trends suggest that Formulation B1 holds promise as a nutritionally improved biscuit alternative worthy of future investigation.

### 3.7. Sensory Analysis

A sensory ranking procedure was conducted according to ISO 8587 (1988) [49] to assess consumer preferences among the biscuit formulations. This forced-choice test evaluates the intensity of sensory attributes across multiple products [50]. In this study, sensory evaluations were performed on four new biscuit formulations containing 25% and 50% rice bran. Acceptance levels were assessed through a preference questionnaire.

The sensory attributes evaluated included aroma intensity, appearance, texture, flavor, and overall quality. The four biscuit formulations were presented in random order. For each sensory attribute—appearance, texture, flavour, and aroma—the trained panel of 12 panelists ranked the biscuits based on intensity for each attribute, from best to worst on a scale of 1 to 4, with 4 representing the highest intensity (best) and 1 representing the lowest intensity (worst). A global assessment was also included, where the panelists provided an overall judgment of the biscuits. The data obtained from the sensory evaluations were analyzed using the non-parametric Friedman test to interpret the organoleptic results.

A trained panel evaluated the four biscuit samples, which were presented to the evaluators in a random order.

The results for the formulations containing 25% and 50% rice bran, from both the Ariete and Type III varieties, provide valuable information on the acceptability of rice-based biscuits with varying bran contents. Additionally, it highlights how the aromatic qualities of the Type III variety, with its distinct pasting properties compared to Ariete, influence sensory preferences. The objective of this analysis was to determine which of the four new formulations would be most preferred.

**Figure 6:**
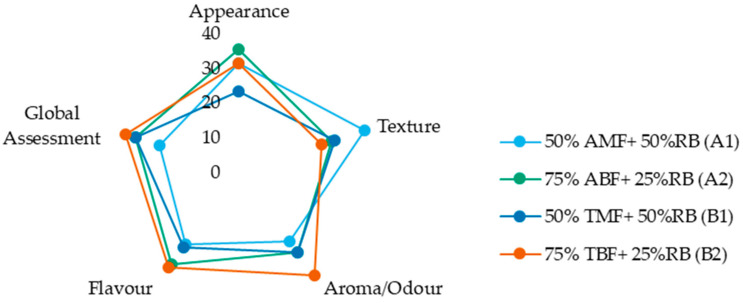
Sum of ranks of each sensory attribute (appearance, texture, aroma, flavour) and global assessment evaluated by the trained panel. AMF—Ariete milled flour, ABF—Ariete brown flour, TMF—Type III milled flour, TBF—Type III brown flour, RB—rice bran.

Figure 6 displays the sum of ranks for the four formulations across the attributes of appearance, texture, aroma (odour), flavour (taste), and overall assessment. While the figure provides a visual summary of relative preferences, analysis of the sensory evaluation data revealed no significant differences among the biscuit formulations for any of the evaluated sensory attributes, as indicated by both the Friedman and Iman–Davenport tests (Table S4 of Supplementary Material). These results suggest that varying the proportions of rice bran and its associated flour and bran fractions does not lead to perceptible differences in appearance, texture, flavour, aroma, or overall preference among the biscuit formulations. This finding is encouraging, as it suggests that incorporating up to 50% rice bran—using different combinations of flour and bran fractions—does not adversely affect the sensory quality of the biscuits, which supports the potential for developing nutritionally enhanced products without compromising consumer acceptability.

## 4. Conclusions

This study demonstrated that incorporating rice bran into biscuit formulations reduced the estimated glycemic index (eGI) while enhancing nutritional and bioactive properties, offering a potentially healthier alternative to traditional biscuits. Using two rice varieties, Ariete (Long A type) and Type III (Basmati type), the results indicated that Type III formulations, characterized by a lower eGI and higher resistant starch (RS) content, may be a more suitable choice for health-conscious consumers. Rice bran enrichment led to a reduction in starch content while increasing protein and lipid levels in the biscuits. Additionally, it improved the concentration of bioactive compounds, particularly oryzanol (ORY) and phytic acid (PA). Notably, biscuits enriched with rice bran exhibited higher levels of these bioactive compounds and a lower eGI compared to commercially available wellness biscuits. Furthermore, sensory analysis revealed no significant differences between the biscuit formulations, suggesting that the nutritional improvements did not negatively impact consumer acceptance. Overall, the results highlight the potential of rice bran-enriched biscuits as a functional and nutritious option, particularly for individuals seeking products with a lower glycemic response. The trends indicate that the formulation with 50% Type III milled flour and 50% rice bran (B1) shows promise as a nutritionally enhanced biscuit alternative, featuring significantly lower eGI, RDS, and TS—beneficial for glycemic control—alongside significantly higher levels of phytic acid (PA) and total antioxidant activity (ORY).

However, it is important to note that this study was conducted on a limited sample size, and further research is needed to confirm these findings across a broader range of formulations and consumer groups. Future studies should explore the long-term health effects of rice bran-enriched biscuits and assess their impact in real dietary settings.

## Figures and Tables

**Table 5:** Physicochemical properties, starch digestion parameters and bioactive compounds in wellness biscuit and Type III biscuits formulations.

Biscuit Formulation			Starch Digestion Parameters			Physicochemical Parameters			Bioactive Compounds		
		eGI	RDS (%)	SDS (%)	RS (%)	TS (%)	FAT (%)	PRTD (%)	PA (g/100 g)	ORY (mg/100 g)	GABA (mg/100 g)
**Wellness Biscuit**	WB	64.98 b	20.76 a	17.50 b	0.38 b	43.95 b	19.54 a	10.83 a	0.70 c	5.44 b	18.79 a
**100% TMF (control)**	B0	66.23 a	19.87 ab	22.51 a	0.91 a	47.38 a	11.63 d	5.70 c	0.25 d	0.92 b	12.50 c
**50% TMF + 50% RB**	B1	57.06 d	12.87 c	14.97 bc	0.34 b	30.07 d	18.10 b	6.49 b	1.96 a	78.78 a	14.05 bc
**75% TBF + 25% RB**	B2	60.59 c	18.24 b	13.61 c	0.33 b	36.91 c	15.34 c	6.31 b	1.59 b	52.71 a	16.23 ab

eGI—estimated glycaemic index; RDS—rapidly digestible starch; SDS—slowly digestible starch; RS—resistant starch; TS—total starch content; FAT—fat content; PRTD—protein content PA—phytic acid; ORY—γ-oryzanol, GABA—γ-aminobutyric acid. TMF—Type III milled flour, TBF—Type III brown flour, RB—rice bran. Values in the same column with different letters are significantly different at *p* ≤ 0.05 by HSD Tukey test.

## Data Availability

Datasets obtained in this study were deposited in the Biodata.pt under the free accession link: https://doi.org/10.34636/DMPortal/RBSZ9K.

## Supplementary Materials

The following supporting information can be downloaded at https://www.mdpi.com/article/10.3390/foods14132276/s1, Table S1: Dunn test differences—Means of eGI and starch digestion parameters, physicochemical properties, bioactive compounds and texture properties for each rice biscuits formulations; Table S2: Tukey test differences—Means of eGI and starch digestion parameters, physicochemical properties and bioactive compounds in wellness biscuit and Type III biscuits formulations; Table S3: Dunn test differences—Means of eGI and starch digestion parameters, physicochemical properties and bioactive compounds in Wellness biscuit and Type III biscuits formulations; Table S4: Sensory analysis—Rank Sums. Friedman statistic test and Iman-Davenport Test for each sensory attribute; Table S5: Tukey test differences—Means of eGI and starch digestion parameters, physicochemical properties, bioactive compounds and texture properties for each rice biscuits formulations.
