# Supplementary material for: Development and Evaluation of Gluten-Free Rice Biscuits: Impact on Glycaemic Index and Bioactive Compounds
Source: Foods. 2025 Jun 26;14(13):2276. doi: 10.3390/foods14132276 (PMC12249346; doi:10.3390/foods14132276)
Supplement: Supplementary file 1 [file foods-14-02276-s001.zip › foods-3691959-supplementary.pdf]

Table S1. Dunn test differences - Means of eGI and starch digestion parameters, physicochemical properties, bioactive compounds and texture properties for each rice biscuits formulations

	Biscuit Formulation	eGI	RDS (%)	SDS (%)	RS (%)	TS (%)	FAT (%)	PRTD (%)	PA (g/100g)	ORY (mg/100g)	GABA (mg/100g)	HARD (N)	SR
A0	100% AMF	66.95 a	24.69 a	16.94 ab	0.22 ab	45.68 ab	12.04 ab	4.20 c	0.16 b	0.20 b	6.78 b	168.30 a	5.90 ab
A1	50% AMF+ 50%RB	60.31 ab	21.18 a	9.89 b	0.19 ab	33.38 bc	18.22 a	6.09 abc	1.90 a	86.56 a	12.03 ab	80.33 ab	5.03 ab
A2	75% ABF+ 25%RB	62.75 ab	20.85 ab	14.59 ab	0.15 b	38.42 abc	16.18 ab	5.27 bc	1.67 ab	66.81 ab	12.08 ab	54.55 b	4.81 ab
B0	100% TMF	66.23 a	19.87 ab	22.51 a	0.91 a	47.38 a	11.63 b	5.70 abc	0.25 ab	0.92 ab	12.50 ab	60.83 ab	6.35 a
B1	50% TMF+ 50%RB	57.06 b	12.87 b	14.97 ab	0.34 ab	30.07 c	18.10 a	6.49 a	1.96 a	78.78 ab	14.05 ab	65.76 ab	4.57 b
B2	75% TBF+ 25%RB	60.59 ab	18.24 ab	13.61 b	0.33 ab	36.91 abc	15.34 ab	6.31 a	1.59 ab	52.71 ab	16.23 a	79.71 ab	4.68 b

Table S2. Tukey test differences – Means of eGI and starch digestion parameters, physicochemical properties, and bioactive compounds in Wellness biscuit and Type III biscuits formulations

	Biscuit Formulation	eGI	RDS (%)	SDS (%)	RS (%)	TS (%)	FAT (%)	PRTD (%)	PA (g/100g)	ORY (mg/100g)	GABA (mg/100g)
WB	Wellness Biscuit	64.98 b	20.76 a	17.50 b	0.38 b	43.95 b	19.54 a	10.83 a	0.70 c	5.44 b	18.79 a
B0	100% TMF	66.23 a	19.87 ab	22.51 a	0.91 a	47.38 a	11.63 d	5.70 c	0.25 d	0.92 b	12.50 c
B1	50% TMF+ 50%RB	57.06 d	12.87 c	14.97 bc	0.34 b	30.07 d	18.10 b	6.49 b	1.96 a	78.78 a	14.05 bc
B2	75% TBF+ 25%RB	60.59 c	18.24 b	13.61 c	0.33 b	36.91 c	15.34 c	6.31 b	1.59 b	52.71 a	16.23 b

Table S3. Dunn test differences – Means of eGI and starch digestion parameters, physicochemical properties and bioactive compounds in wellness biscuit and Type III biscuits formulations

	**Biscuit Formulation**	**eGI**	**RDS (%)**	**SDS (%)**	**RS (%)**	**TS (%)**	**FAT (%)**	**PRTD (%)**	**PA (g/100g)**	**ORY (mg/100g)**	**GABA (mg/100g)**
WB	Wellness Biscuit	64.98 ab	20.76 a	17.50 ab	0.38 a	43.95 ab	19.54 a	10.83 a	0.70 ab	5.44 ab	18.79 a
B0	100% TMF	66.23 a	19.87 ab	22.51 a	0.91 a	47.38 a	11.63 b	5.70 b	0.25 b	0.92 b	12.50 b
B1	50% TMF+ 50%RB	57.06 b	12.87 b	14.97 ab	0.34 a	30.07 b	18.10 ab	6.49 ab	1.96 a	78.78 a	14.05 ab
B2	75% TBF+ 25%RB	60.59 ab	18.24 ab	13.61 b	0.33 a	36.91 ab	15.34 ab	6.31 ab	1.59 ab	52.71 a	16.23 b

Table S4. Sensory analysis – Rank Sums. Friedman statistic test and Iman-Davenport Test for each sensory attribute

	**Biscuit Formulation**	**Appearance**	**Texture**	**Aroma/Odour**	**Flavour**	**Global Assessment**
A1	50% AMF+ 50%RB	31	38	25	26	24
A2	75% ABF+ 25%RB	35	28	29	33	31
B1	50% TMF+ 50%RB	23	29	29	27	31
B2	75% TBF+ 25%RB	31	25	37	34	34
	**Friedman Test** (Chi-Square-approximation)	3.8	4.7	3.8	2.5	2.7
	p-value	0.2839	0.1951	0.2839	0.4753	0.4402
	**Iman-Davenport Test** (F-approximation)	1.2981	1.6518	1.2981	0.8209	0.8919
	p-value	0.2914	0.1964	0.2914	0.4917	0.4556

Table S5. Tukey test differences - Means of eGI and starch digestion parameters, physicochemical properties, bioactive compounds and texture properties for each rice biscuits formulations

	Biscuit Formulation	eGI	RDS (%)	SDS (%)	RS (%)	TS (%)	FAT (%)	PRTD (%)	PA (g/100g)	ORY (mg/100g)	GABA (mg/100g)	HARD (N)	SR
A0	100% AMF	66.95 a	24.69 a	16.94 b	0.22 bc	45.68 a	12.04 d	4.20 e	0.16 c	0.20 c	6.78 b	168.30 a	5.90 b
A1	50% AMF+ 50%RB	60.31 c	21.18 b	9.89 d	0.19 c	33.38 c	18.22 a	6.09 b	1.90 a	86.56 a	12.03 a	80.33 b	5.03 c
A2	75% ABF+ 25%RB	62.75 b	20.85 b	14.59 bc	0.15 c	38.42 b	16.18 b	5.27 c	1.67 b	66.81 ab	12.08 a	54.55 c	4.81 cd
B0	100% TMF	66.23 a	19.87 bc	22.51 a	0.91 a	47.38 a	11.63 d	5.70 d	0.25 c	0.92 c	12.50 a	60.83 bc	6.35 a
B1	50% TMF+ 50%RB	57.06 d	12.87 d	14.97 bc	0.34 b	30.07 d	18.10 a	6.49 a	1.96 a	78.78 a	14.05 a	65.76 bc	4.57 d
B2	75% TBF+ 25%RB	60.59 c	18.24 c	13.61 c	0.33 b	36.91 b	15.34 c	6.31 ab	1.59 b	52.71 b	16.23 a	79.71 b	4.68 cd
